# Assessment of psychotropic medicines utilization patterns for the treatment of mental and neurological disorders in Public Hospitals of Hadiya Zone, Southern Ethiopia: A record-based study

**DOI:** 10.1371/journal.pmen.0000665

**Published:** 2026-08-07

**Authors:** Mengistu Girma Tirore, Teferi Gedif Fenta, Alemayehu Adnew Teshale

**Affiliations:** 1 Department of Pharmacy, College of Health Sciences, Wachemo University, Hossana, Ethiopia; 2 School of Pharmacy, College of Health Sciences, Addis Ababa University, Addis Ababa, Ethiopia; PLOS: Public Library of Science, UNITED KINGDOM OF GREAT BRITAIN AND NORTHERN IRELAND

## Abstract

Mental and neurologic disorders are a growing global health concern. Psychotropic medicines are vital for the treatment of these disorders, but issues such as inappropriate prescribing and polypharmacy are prevalent, especially in low- and middle- income countries like Ethiopia. This study assessed psychotropic medicine use patterns in public hospitals of Hadiya zone, Southern Ethiopia. A facility-based cross-sectional study was conducted from June 15 to December 30, 2019, involving the review of 1200 patients medical charts. Data were collected by reviewing the medical charts of patients with mental and neurologic disorders using a data abstraction format. Data were entered and analyzed using SPSS version 26. The results showed that, the most commonly treated mental and neurologic disorders were psychosis (27.50%), followed by schizophrenia (15.67%), epilepsy (13%), and depression (11.92%). Antipsychotics (48.1%) and antidepressants (25.7%) were the most commonly used psychotropic medicines. Chlorpromazine, haloperidol, and thioridazine were the top three most prescribed antipsychotics which constituted 71.34% of antipsychotics use in public hospitals of Hadiya zone. The most commonly prescribed combinations were antipsychotics with antidepressants and antipsychotics with anti-anxiety medicines. Typical antipsychotics were prescribed more often (73.86%) than atypical antipsychotics (26.14%). Among antidepressants, amitriptyline, a tricyclic antidepressant, was most frequently prescribed (74.9%), followed by fluoxetine, selective serotonin reuptake inhibitor (21.5%). Treatment shift was undertaken in 11.5% of patients. The percentage of encounters with two or more psychotropic medicines was 36.1%, which shows a polypharmacy practice. Antipsychotics were the most commonly utilized psychotropic medicines, followed by antidepressants and anticonvulsants. Chlorpromazine, Amitriptyline, and Risperidone showed a higher proportion of utilized medicines. More than one-third of psychiatric patients were prescribed psychotropic polypharmacy. Close to 10% of psychiatric patients had a treatment switch, mostly because of poor improvement, relapse, side effects, and non-adherence. Further prospective continuous studies on both prevalence and use patterns need to be undertaken to get more information.

## Introduction

Mental, neurological, and substance use disorders (MNS) are becoming a public health priority and major causes of morbidity. They are usually associated with significant distress in social, economic, or other important activities. Research findings indicated that 14% of the world’s disease burden measured in disability-adjusted life years (DALYs) can be attributed to MNS disorders, with a disproportionately high percentage in low and middle income countries (LMICs). This burden, a treatment gap of more than 75%, results from the fact that many LMICs devote less than 2% of their overall healthcare budgets to mental health [1].

Worldwide, more than 792 million people were diagnosed with mental health problems in 2017. This is somewhat more than one in ten people worldwide (10.7%) [2]. Around 80% of persons who have had a mental illness at some point in their lives come from low- and middle-income nations [3,4]. However, these nations paid little attention to important health issues [5]. To address the demand for healthcare services, mental healthcare services are integrated into the primary healthcare system [6].

In Ethiopia, mental disorders are becoming among the top non-communicable disorders in terms of burden. It accounts for about 4.0% of the country’s overall disease burden [7]. The burden of mental health problems, mainly in rural Ethiopia, constituted 11% of the total disease burden, with schizophrenia and depression included in the top ten most burdensome health problems lists [8]. The prevalence of mental disorders was 18% for adults and 15% for children [9]. Studies conducted in Hadiya and Kambata adult communities showed that the prevalence of mental illness was 17.2%, with 60% male and 40% female proportionality. Stress and family history were the most determining factors recognized [10].

Psychotropic Medicines (PM) are widely used to treat mental and neurologic disorders, and their major impacts are on motor functions. These medicines not only improve the conditions of patients but also are known to cause long-term, potentially harmful adverse effects unless used appropriately [11].

Although there is a discrepancy between standards and prescribing practices for commonly prescribed medications like antidepressants and antipsychotics, following guidelines may lower the risk. Use of multiple antipsychotic medications, administration of dosages higher than recommended levels, use of psychotropic medications other than antipsychotics, use of antidepressants without a valid reason, and/or use of olanzapine—an antipsychotic that is particularly likely to cause significant weight gain—were the most prevalent practices that did not comply with guidelines. This is frequently observed in the treatment of mental disorders like schizophrenia, in which combinations of psychotropic medicines are commonly utilized [12]. Psychotropic combination treatment may include combinations of different antipsychotic medicines or combinations of antipsychotics with antidepressants, mood stabilizers, or anxiolytics/hypnotics [13].

Study findings reported diverse patterns of psychotropic medication utilization. Even though there are more recent medications with better side effect profiles, first-generation antipsychotics like haloperidol and chlorpromazine are still commonly administered in low- and middle-income nations because they are more affordable. Comorbidities and complex diagnoses can lead to polypharmacy, which raises concerns regarding non-compliance and harmful drug reactions [14]. Psychotropic polypharmacy is typically defined as the use of two or more psychotropic drugs in the same patient or the treatment of the same problem with two or more drugs (of the same chemical class or pharmacologic activities) [15].

Psychotropic medicines utilization studies around the world have revealed widespread irrational prescribing, dispensing, and use practices [16–18]. Psychotropic medicines polypharmacy is a critical problem in psychiatric treatment practice, and it could put users of medicines at risk of drug interactions, side effects, and unintended effects [18,19]. Research findings demonstrated that despite progress in psychopharmacology and a better understanding of mental health problems treatment, the use of psychotropic polypharmacy is escalating. The frequency of prescribing polypharmacy in psychiatry ranges from 13% to 90%. Managing polypharmacy requires an understanding of its associated factors. Structuring education, clinical guidelines, and decision algorithms for the suitable management of different conditions are compelling ways to manage unreasonable polypharmacy [20]. Another study in Nigeria indicated polypharmacy was in 92.3%, of which 62.2% of clients were on antipsychotic/anticholinergic combinations [18].

According to a study conducted in the Amanuel Mental Specialized Hospital, the prevalence of irrational antipsychotic medicine utilization was 32.6% [17]. Another research done at Nekemte Referral Hospital revealed that more than half of the prescriptions (52.21%) contained two psychotropic medicines [21]. Patients experiences with psychotropic medicines during the treatment may have a long-lasting effect on how they feel about the medicine and the course of illness. Even though there are a few studies conducted in different health facilities nationally, there is no adequate information on the patterns of psychotropic medicines prescribing among mental and neurologic disorder patients in the current study area. Therefore, a current study was conducted to assess psychotropic medicines utilization patterns in Hadiya Zone Public Hospitals.

## Methods and materials

### Ethics statement

The study was reviewed and approved by the Addis Ababa University, College of Health Science, School of Pharmacy Ethical Review Board (ERB) (ERB/SOP/105/06/2019). According to national legislation and institutional policies, written informed consent from participants or participants’ legal guardians was not necessary for the involvement in the study. Besides, the study involves a retrospective review of de-identified patients’ charts, with no direct contact or intervention. Since obtaining informed consent was impractical and posed no additional risk to patients, it was not required. The study was conducted in accordance with the principles of the Declaration of Helsinki.

### Study design and setting

A facility-based cross facility-based cross sectional study was conducted in Public Hospitals of Hadiya Zone. Hadiya Zone is one of the 14 Zones in the Southern Nations, Nationalities and Peoples Regions of Ethiopia. The Zone had four public hospitals. These are Homacho Primary Hospital (HPH), Shone Primary Hospital (SPH), and Wachemo University Nigist Eleni Mohammed Memorial Comprehensive Specialized Hospital (WUNEMMCSH) [Hadiya Zone Health Bureau report, 2019]. WUNEMMCSH has two psychiatrists and three psychiatry nurses, whereas SPH and HPH started an outpatient psychiatry service in 2017 with two psychiatry nurses. Primary mental healthcare providersinclude psychiatrists and psychiatric nurses. The diagnosis is based on the Diagnostic and Statistical Manual of Mental Disorders (DSM)-5 (American Psychiatric Association, 2013). The diagnostic practice was consistent across three hospitals. The study period for data collection was from June 15 to December 30 2019.

### Study population and sample size

A census approach was employed for this study. All patients’ records meeting the eligibility criteria during the study period were reviewed and included in the analysis. Since the total number of eligible records was relatively small and manageable, no sample size calculation or sampling procedures were performed. Furthermore, given that the patients’ medical records were paper-based and susceptible to loss or incompleteness, inclusion of all available records was necessary to maximize the sample size and reduce potential selection bias. A total of 1200 patients’ records were identified and reviewed, and all were included in the final analysis. Diagnoses recorded in the psychiatry outpatient clinics were classified as either mental disorders or neurologic disorders. Neurologic disorders included epilepsy, seizure, headache and dementia, whereas other listed disorders fall under mental disorders.

### Inclusion and exclusion criteria

All medical charts of patients diagnosed and treated with mental and neurologic disorders at psychiatry outpatient clinics in public hospitals within Hadiya Zone, from September 2015 to August 2019, were considered. Medical charts were included if they contained complete information on prescribed medicines and relevant clinical characteristics. The patients’ medical charts with missing prescription information, incomplete treatment documentation and duplicate documents were excluded.

### Data collection, management and analysis

Data were collected by reviewing medical charts using a data abstraction form. The data variables include patient demographics, diagnosis, prescribed medicines, dosage, and duration. Data were entered into SPSS version 26 (as attached in S1 Data) and analyzed using SPSS version 26. Descriptive statistics such as frequency, percentage, mean, median, and interquartile range are used to summarize the results.

## Results

### Socio-demographic characteristics of study participants

A total of 1200 patients’ charts were reviewed; of whom 655 (54.6%) were males, 760 (63.3%) aged between 15–29 years. The mean age was 29.5 (SD ± 13.8) years. The male-to-female ratio was 1.2:1 (Table 1).

**Table 1:** Demographic characteristics of the mental and neurologic disorder patients treated from 2015-2019 in Public Hospitals of Hadiya Zone, SNNPR, Ethiopia, 2019.

Hospitals	Characteristics		Years											
			2015		2016		2017		2018		2019		Total	
			N	%	N	%	n	%	N	%	N	%	N	%
WUNEMMCSH	Sex	Male	141	52.4	104	54.7	91	58.0	78	52.7	167	54.8	581	54.3
		Female	128	47.6	86	45.3	66	42.0	70	47.3	138	45.2	488	45.7
		Total	269	100	190	100	157	100	148	100	305	100	1069	100
	Age	15-29	171	63.6	120	63.2	101	64.3	94	63.5	190	62.3	676	63.2
		30 – 64	86	32.0	59	31.1	51	32.5	52	35.1	103	33.8	351	32.8
		65+	12	4.5	11	5.8	5	3.2	2	1.4	12	3.9	42	3.9
		Total	269	100	190	100	157	100	148	100	305	100	1069	100
SPH	Sex	Male					22	42.3	5	50.0	4	66.7	31	45.6
		Female					30	57.7	5	50.0	2	33.3	37	54.4
	Age	15-29					32	61.5	9	90.0	4	66.7	45	66.2
		30 – 64					20	38.5	1	10.0	2	33.3	23	33.8
		65+					0	0.0	0	0.0	0	0.0	0	0.0
		Total	–	–	–	–	52	100	10	100	6	100	68	100
HPH	Sex	Male					8	61.5	22	64.7	13	81.2	43	68.3
		Female					5	38.5	12	35.3	3	18.8	20	31.7
	Age	15-29					11	84.6	21	61.8	7	43.8	39	61.9
		30 – 64					2	15.4	10	29.4	9	56.2	21	33.3
		65+					0	0.0	3	8.8	0	0.0	3	4.8
		Total	–	–	–	–	13	100	34	100	16	100	63	100

Note: Until 2017, there was no separate mental and neurologic disorders treatment service in SPH and HPH at psychiatry outpatient clinics level.

In WUNEMMCSH, psychosis has shown an increase in proportion from 36.4% in 2015 to 42.4% in 2017 and then a decrease in 2018 and 2019, whereas schizophrenia has shown an increase throughout the study period, from 5.6% in 2015 to 23.9% in 2019. The proportion of patients treated for depression increased from 9.3% in 2015 to 20.7% in 2019. In SPH and HPH, a decreasing trend in the proportion of epilepsy patients was observed from 86.5% in 2017 to 16.7% in 2019 in SPH and 53.8% in 2017 to 31.3% in 2019 in HPH (Table 2).

**Table 2:** Trends of mental and neurologic disorder patients over the five years (2015-2019) in Hadiya zone public Hospitals, SNNPR, Ethiopia, and 2019.

Hospitals	Diagnosis	Years											
		2015		2016		2017		2018		2019		Total	
		n	%	N	%	n	%	n	%	n	%	n	%
WUNEMMCSH	Relapsed psychosis	14	5.2	14	7.3	16	10.3	6	4.1	11	3.6	61	5.7
	Psychosis	84	31.2	40	20.9	50	32.1	30	20.3	49	16.1	253	23.7
	Schizophrenia	15	5.6	19	9.9	30	19.2	42	28.4	73	23.9	179	16.7
	Bipolar I	9	3.3	20	10.5	21	13.5	20	13.5	33	10.8	103	9.6
	Depression	25	9.3	16	8.4	12	7.7	14	9.5	63	20.7	130	12.2
	Anxiety	42	15.6	6	3.1	14	9.0	17	11.5	16	5.2	95	8.9
	Epilepsy	41	15.2	41	21.5	0	0.0	0	0.0	0	0.0	82	7.7
	Seizure	1	0.4	3	1.6	0	0.0	0	0.0	0	0.0	4	0.4
	Others*	38	14.1	32	16.8	13	8.3	19	12.8	60	19.7	162	15.2
	Total	269	100	191	100	156	100	148	100	305	100	1069	100
SPH	Psychosis					2	3.8	4	40.0	4	66.7	10	14.7
	Schizophrenia					4	7.7	1	10.0	0	0.0	5	7.4
	Bipolar I					0	0.0	0	0.0	0	0.0	0	0.0
	Depression					0	0.0	0	0.0	1	16.7	1	1.5
	Anxiety					1	1.9	0	0.0	0	0.0	1	1.5
	Epilepsy					45	86.5	5	50.0	1	16.7	51	75.0
	Seizure					0	0.0	0	0.0	0	0.0	0	0.0
	Others*					0	0.0	0	0.0	0	0.0	0	0.0
	Total	–	–	–	–	52	100	10	100	6	100	68	100
HPH	Psychosis					1	7.7	5	14.7	0	0.0	6	9.5
	Schizophrenia					1	7.7	2	5.9	1	6.2	4	6.3
	Bipolar I					2	15.4	1	2.9	0	0.0	3	4.8
	Depression					0	0.0	4	11.8	8	50.0	12	19.0
	Anxiety					0	0.0	0	0.0	0	0.0	0	0.0
	Epilepsy					7	53.8	11	32.4	5	31.3	23	36.5
	Seizure					1	7.7	7	20.6	0	0.0	8	12.7
	Others*					1	7.7	4	11.8	2	12.5	7	11.1
	Total	–	–	–	–	13	100	30	100	16	100	56	100

Others*: Posttraumatic stress disorder, suicide attempt, mood disorders, headache, Dementia, delusion, Bipolar II, insomnia, bipolar, and depression with psychotic features.

Note: Epilepsy, seizure, headache and dementia are neurologic disorders, whereas other listed disorders fall under mental disorders.

Regarding sex and age distribution of patients treated over the five years (2015–2019), the proportion of male-to-female psychosis patients was equal, with 165 (50%) in each group, whereas the proportion of males with schizophrenia was higher than that of females (56.4% versus 43.6%). The proportion of depression was higher in males than in females (55.2% versus 44.8%). The majority of psychosis cases, 210 (63.6%), were in the age group 15–29 (Table 3).

**Table 3:** Mental and neurologic disorders seen in Hadiya zone public hospitals, SNNPR, Ethiopia over the period (2015 to 2019) by sex and age group, 2019.

Diagnosis of patients	Sex of patient			Age of the patient			
	Male	Female	Total	15-29	30 – 64	65+	Total
	n(%)	n(%)	n(%)	n(%)	n(%)	n(%)	n(%)
Relapsed psychosis	29(47.5)	32(52.5)	61(100)	34(55.7)	25(41)	2(3.3)	61(100)
Psychosis	136(50.6)	133(49.4)	269(100)	176(65.4)	85(31.6)	8(3)	269(100)
Schizophrenia	106(56.4)	82(43.6)	188(100)	119(63.3)	64(34)	5(2.7)	188(100)
Bipolar I	60(56.6)	46(43.4)	106(100)	88(83)	18(17)	0	106(100)
Depression	79(55.2)	64(44.8)	143(100)	93(65)	44(30.8)	6(4.2)	143(100)
Anxiety	57(59.4)	39(40.6)	96(100)	45(46.9)	45(46.9)	6(6.2)	96(100)
Epilepsy	78(50)	78(50)	156(100)	114(73.1)	39(25)	3(1.9)	156(100)
Seizure	5(41.7)	7(58.3)	12(100)	8(66.7)	4(33.3)	0	12(100)
Others*	105(62.1)	64(37.9)	169(100)	83(49.1)	71(42)	15(8.9)	169(100)
Total	655(54.6)	545(45.4)	1200(100)	760(63.3)	395(32.9)	45(3.8)	1200(100)

### Medication utilization patterns

Regarding psychotropic medicine utilization, the most commonly prescribed medicine categories in WUNEMMCSH were antipsychotics 793 (51.2%), followed by antidepressants 428 (27.6%) and anticonvulsants 207 (13.4%). In SPH and HPH, anticonvulsants were the most commonly utilized medicines, 48 (68.6%) and 33 (42.3%), respectively. In WUNEMMCSH, utilization of antipsychotics showed an increase from 184 (46.9%) in 2015 to 241 (54.4%) in 2019, whereas consumption of anticonvulsants showed a decrease from 55 (14%) in 2015 to 37 (8.4%) in 2019. Anticonvulsant use also showed a decrease in the two primary hospitals from 2017 to 2019 (Table 4).

**Table 4:** Psychotropic Medicines consumed during the five years (2015-2019) in Hadiya zone public hospitals, SNNPR, Ethiopia, and 2019.

Hospitals	Medicines categories	Years											
		2015		2016		2017		2018		2019		Total	
		N	%	N	%	N	%	N	%	n	%	n	%
WUNEMMCSH	Antianxiety	27	7.0	17	6.0	16	7.0	8	4.0	54	12.2	122	7.8
	Anticonvulsant	55	14.0	67	23.5	23	10.0	25	12.5	37	8.4	207	13.4
	Antidepressants	126	32.1	73	25.6	62	27.0	56	28.0	111	25.0	428	27.6
	Antipsychotics	184	46.9	128	44.9	129	56.0	111	55.5	241	54.4	793	51.2
	Total	392	100	285	100	230	100	200	100	443	100	1550	100
SPH	Antianxiety					1	1.9	1	9.1	0	0.0	2	2.9
	Anticonvulsants					44	83.0	3	27.3	1	16.7	48	68.6
	Antidepressants					2	3.8	0	0.0	1	16.7	3	4.3
	Antipsychotics					6	11.3	7	63.6	4	66.6	17	24.2
	Total	–	–	–	–	53	100	11	100	6	100	70	100
HPH	Antianxiety					1	5.3	3	8.1	2	9.1	6	7.7
	Anticonvulsants					10	52.6	18	48.7	5	22.7	33	42.3
	Antidepressants					1	5.3	5	13.5	9	40.9	15	19.2
	Antipsychotics					7	36.8	11	29.7	6	27.3	24	30.8
	Total	–	–	–	–	19	100	37	100	22	100	78	100

Overall, the most commonly prescribed category of psychotropic medicines was antipsychotics 834 (48.1%), followed by antidepressants 446 (25.7%) and anticonvulsants or mood stabilizers 288 (16.6%). Among the psychotropic medicines, the most commonly utilized antipsychotics were Chlorpromazine 259 (15%), followed by Risperidone 216 (12.5%) and Haloperidol 198 (11.4%). Among antidepressants, amitriptyline, Tricyclic Antidepressants (TCA), was most frequently prescribed (74.9%), followed by fluoxetine, Selective Serotonin Reuptake Inhibitor (SSRI) (21.5%) (Table 5).

**Table 5:** Psychotropic medicines prescribed during the five years (2015-2019) in Hadiya Zone Public Hospitals, SNNPR, Ethiopia, 2019.

Psychotropic medicines categories	Psychotropic medicines	n	n %	Total n %
Antianxiety	Diazepam	64	49.2	3.7
	Diazepam injection	66	50.8	3.8
	Total	130	100.0	7.5
Anticonvulsants/mood stabilizers	Carbamazepine	52	18.1	3.0
	Phenobarbitone	148	51.4	8.5
	Phenytoin	20	6.9	1.2
	Sodium valproate	68	23.6	3.9
	Total	288	100.0	16.6
Antidepressants	Amitriptyline	334	74.9	19.2
	Fluoxetine	96	21.5	5.5
	Imipramine	16	3.6	0.9
	Total	446	100.0	25.7
Antipsychotics	Chlorpromazine	259	31.0	15.0
	Fluphenazine injection	21	2.5	1.2
	Haloperidol	198	23.7	11.4
	Haloperidol injection	64	7.7	3.7
	Risperidone	216	26.0	12.5
	Thioridazine	74	8.9	4.3
	Olanzapine	2	0.2	0.1
	Total	834	100.0	48.1
Anticholinergic	Benzhexol	36	100.0	2.1
Total Psychotropic medicines prescribed		1734	100.0	100.0

From the total psychotropic medicines prescribed, the most commonly prescribed combinations were chlorpromazine with amitriptyline and haloperidol injection with diazepam injection (Table 6).

**Table 6:** Combination psychotropic medicines prescribed during the period 2015-2019 in Hadiya Zone Public Hospitals, SNNPR, Ethiopia, 2019.

Psychotropic medicine	Chlorpromazine	Risperidone	Phenobarbitone	Amitriptyline	Na. Val	Haloperidol	Carbamazepine	Thioridazine	Fluoxetine	Diazepam
Chlorpromazine	**259**	**216**	**148**							
Amitriptyline	60	15	1	**334**						
Na. Val	8	28	1	0	68					
Haloperidol	2	1	3	40	10	**198**				
Carbamazepine	9	1	3	0	0	28	**52**			
Thioridazine	1	0	0	27	0	1	0	**74**		
Fluoxetine	9	30	1	0	2	10	0	0	**96**	
Diazepam	2	30	1	1	8	15	0	1	11	**64**
Imipramine	3	2	0	0	0	3	0	1	0	0
Hal inj	26	9	0	6	3	21	5	3	1	5
Diazepam inj	26	10	0	6	3	22	5	3	1	5
Benzhexol	11	2	0	6	3	19	2	2	1	0
Fluphenazine inj	2	2	0	3	1	0	0	0	1	1

Hal inj- Haloperidol injection, Na. val-Sodium valproate.

Note: Diagonal cells represent the total number for each medicine (e.g., Chlorpromazine = 259, Amitriptyline = 334). Off–diagonal cells indicate the number of cases where both medicines were prescribed together as co-prescriptions. Each off-diagonal value, therefore, reflects the frequency of co-prescription of the two corresponding medicines.

In WUNEMMCSH, a treatment shift occurred for 139 patients, representing 11.5% of the total. The primary reasons for this shift included inadequate control or improvement with the initial treatment (46 patients, 33.1%), relapse (29 patients, 20.9%), side effects (22 patients, 15.8%), and non-adherence (19 patients, 13.7%) (see Table 7).

**Table 7:** Treatment shift during (2015-2019) at WUNEMMCSH, SNNPR, Ethiopia, 2019.

Hospital	Reasons for treatment shift	Years											
		2015		2016		2017		2018		2019		Total	
		N	%	n	%	n	%	n	%	n	%	N	%
WUNEMMCSH	Due to poor control/no improvement	12	32.4	10	38.5	6	26.1	7	28.0	11	39.3	46	33.1
	Due to relapse	9	24.3	4	15.4	3	13.0	8	32.0	5	17.9	29	20.9
	Due to no adherence	8	21.6	6	23.1	2	8.7	1	4.0	2	7.1	19	13.7
	Due to side effects	3	8.1	3	11.5	5	21.7	5	20.0	6	21.4	22	15.8
	Unavailability of medication/s	3	8.1	0	0.0	2	8.7	1	4.0	0	0.0	6	4.3
	Unknown reason	1	2.7	0	0.0	5	21.7	2	8.0	2	7.1	10	7.2
	Others**	1	2.7	3	11.5	0	0.0	1	4.0	2	7.1	7	5.0
	Total	37	100	26	100	23	100	25	100	28	100.0	139	100
Switch from typical antipsychotics to atypical antipsychotic(only Risperidone)										14.4%			
Switch from typical antipsychotics to typical antipsychotics and other classes*										85.6%			

Of the 1200 medical charts reviewed, the overall average number of psychotropic medicines per encounter was 1.5. The average number of drugs per encounter was 1.53, 1.04, and 1.18 in Wachemo University Hospital, Shone, and Hoomacho Hospital, respectively. The percentage of one, two, three, and four psychotropic medicines prescribed in 1200 medical charts reviewed was 767/1200 (63.9%), 352/1200 (29.3%), 58/1200 (4.8%), and 23/1200 (2%), respectively. The percentage of encounters with psychotropic medicine injections prescribed was 91/1200 (7.6%). Benzodiazepine was prescribed for 131/1200 (11%) of participants.

## Discussion

In the current study, the majority of mental disorders treated over the five years covered were in the 15–29 age group. This age range was common across most mental disorders in other studies [21,22]. Most adult mental disorders have a juvenile onset, and problems persist if timely intervention is not provided [23]. Among the total mental disorders treated in Hadiya Zone public hospitals, psychosis cases constituted 27.5%. Of these, 61 (5.08%) were relapsed psychosis. Underlying causes of relapses may include lack of adherence and inappropriate treatment due to stock-outs of medicines. A study conducted in southwest Ethiopia revealed that the odds of developing relapsed psychosis among treatmentadherent clients were 69% lower than among non-adherent clients [24]. Regarding utilization of psychotropic medicines over the five years at WUNEMMCSH, the most commonly utilized psychotropic medicine categories were antipsychotics (51.2%), followed by antidepressants (27.6%) and anticonvulsants (13.4%). This high antipsychotics utilization suggests that psychosis is a major mental health disorder being treated.

This finding is consistent with the findings of other studies in which antipsychotic medicines are the most commonly utilized psychotropic medicines due to their expanded indications [21,25]. In WUNEMMCSH, utilization of antipsychotics showed an increasing trend, which may be related to increasing cases of psychosis, whereas consumption of anticonvulsants showed a decreasing trend, which might be related to the shift of epilepsy treatment to general outpatient department. Anticonvulsant or mood stabilizer utilization showed a decreasing trend in the two primary hospitals, which mostly offer epilepsy and seizure cases treatment service. This might be related to the limited availability of psychotropic medicines in the hospitals and patients’ low demand for mental health services utilization. A study conducted on epilepsy treatment in a rural part of Ethiopia indicated that, though anticonvulsant medicines are the mainstay of epilepsy treatment, the majority of individuals with epilepsy could not get appropriate treatment [26].

The three frequently prescribed antipsychotic medicines, namely, chlorpromazine, haloperidol, and thioridazine, constituted 595/834 (71.34%) of antipsychotic utilization at Hadiya Zone public hospitals. Similar research findings from Taiwan also indicated that the above three medicines constituted 66%-74% of antipsychotic use [27]. This may be attributed to the limited availability and accessibility of psychotropic medicines, particularly in health facilities located in remote areas. According to the Ethiopian Standard Treatment Guideline, thioridazine has been significantly linked to aberrant electrocardiogram (ECG) readings. Consequently, its use was confined to specialist supervision as second-line treatment of schizophrenia in adults. The patients were recommended for regular follow-up, which includes ECG screening and electrolyte measurement before treatment, after each dose increase and at 6-month intervals. Additionally, the patients undergoing prolonged treatment were monitored for visual impairments.

Among the antipsychotics, chlorpromazine was the most commonly prescribed medicine for patients, 259/834 (31.01%), followed by risperidone, 216/834 (25.9%). This result was lower than the study report from Amanuel Mental Specialized Hospital, which showed psychiatric patients’ frequent prescriptions with chlorpromazine 271 (45.2%), followed by risperidone 154 (25.7%). Another study from Nekemte Referral Hospital indicated 132 (20.95%) patients were prescribed chlorpromazine [17,21].

The current study results depicted that typical antipsychotics were utilized more often 616/834 (73.86%) than atypical antipsychotics 218/834 (26.14%). This result was similar to the findings of two studies in Ethiopia, which revealed preferences for typical antipsychotics over atypical ones [17,21]. A study report from a large state Medicaid program showed that, when using atypical antipsychotics, there was a significant decrease in treatment switching and concurrent medication use compared to those using conventional antipsychotics [28]. However, it was in contrast with the study conducted in India, which showed increased use of atypical antipsychotics (43.83%) compared to typical antipsychotics (26.32%) due to their better safety and tolerability profiles. Another study conducted in a Hungarian psychiatry hospital on schizophrenia outpatients revealed 30% of patients were treated with first-generation antipsychotics and 80% with atypical ones [29,30].

The present study results showed that psychiatry nurses were more accustomed to prescribing conventional psychotropic medicines over non-conventional ones. These might be due to the limited accessibility and availability of psychotropic medicines in the hospitals, their cost affordability for consumers compared to newer atypical antipsychotics. Typical antipsychotics are being replaced by atypical antipsychotics in developed countries over time, but they continue to be utilized foremost in developing nations due to their relative cost affordability compared with atypical antipsychotics [31].

Antidepressants were the second most commonly prescribed medicine category found in the present study, 446 (25.7%). From TCA, amitriptyline was the most commonly prescribed antidepressant (334/446 (78.5%)), followed by fluoxetine from the newer SSRI (98/446 (21.5%)). This result is comparable with the study report from Nekemte Referral Hospital [21]. It is in contrast with a studies from Italy, where SSRIs represented 73% of use, whereas TCAs accounted 27% [32]; France, where 45% of the patients took SSRIs, with fluoxetine leading to 30% of others, and use of TCAs was 39% [33]; and India, with 36.66% SSRI and 21.96% TCA [29]. Even though both antidepressant types have been shown to have similar onsets of action and therapeutic efficacy, SSRIs are generally significantly favored over other antidepressant types. This is because SSRIs have fewer muscarinic, histaminic, and adrenergic side effects and are relatively safer at higher doses that promote better compliance [34]. Hence, SSRIs were recommended as first-line pharmacotherapy for depression [35].

Anticonvulsants/mood stabilizers were the third most commonly consumed medicine class identified in this study, 288/1734 (16.6%), from which phenobarbitone comprised148/288 (51.3%), followed by sodium valproate 68 (23.6%), and carbamazepine 52 (18.1%). Study results reported from Gondor indicated that the most commonly utilized anticonvulsants were phenobarbitone (61.89%), phenytoin (11.27%), and carbamazepine (3.3%) [36]. Another comparable study reported from Jimma indicated that phenobarbitone, which was prescribed in 55% of cases, is a commonly used anticonvulsant [37]. The difference might be due to differences in the availability and affordability of mental health service centres. A study conducted in Taiwan showed that the most frequently prescribed anticonvulsants were carbamazepine, phenytoin, and sodium valproate, with 56.9%, 31.96%, and 30.73%, respectively [36].

The rationale behind the combination of medicines is to boost the effectiveness of monotherapy. In the current study, antipsychotics were combined with antidepressants for 200 mental disorder cases. Chlorpromazine and amitriptyline were the most commonly combined psychotropic medicines followed by haloperidol with amitriptyline and risperidone with fluoxetine. This indicates that both psychosis and depression are common comorbidities in psychiatry clinics. Combinations of TCAs and antipsychotic medicines in individuals with psychotic depression are effective but should be administered initially at low doses and gradually. Because of the suppression of their metabolism arising from their pharmacological activity, the two classes are considered to have sedative and anticholinergic effects that may be prolonged and enhanced when used in combination. The concomitant use of more than one psychotropic medicine needs to be reviewed to improve the quality and safety of patients. It also needs caution when increasing or withdrawing the medications to avoid the risk of seizures that may be increased by lowering the seizure threshold [38].

Clinically, switching from one antipsychotic to another antipsychotic may be indicated when there is a symptom of unsatisfactory response or adverse effect and ineffective treatment. In the present study, treatment change occurred in 139/1200 (11.5%) patients, and the main reasons for the shift were inadequate control/improvement, relapse, side effects, and non-adherence. Study results reported from Indian psychiatry hospital indicated that antipsychotic treatment changes were made in 26% of patients, and the stated justification was lack of efficacy with prior medicine, stock-out of medicines, adverse drug reaction, and high cost of medicines [39]. Another study finding reported from the United States of America demonstrated that the underlying reasons mentioned for treatment switching were the ineffectiveness of previous treatment, side effects, and other or unknown reasons [40]. A study conducted in Turkey indicated that 60.4% of antipsychotic changes occurred due to lack of efficacy with prior antipsychotics, intolerability with given medicines, lack of adherence, and stock-out of psychotropic medicines. Lack of effectiveness and clients’ requests for treatment change were mostly observed with chlorpromazine treatment. Since chlorpromazine was commonly utilized in the current study area, it might be the reason for most treatment changes. Switching from typical antipsychotics to atypical antipsychotics (only risperidone) was only 14.4%, whereas the remaining switching was undertaken from one typical antipsychotic to another typical antipsychotic, mainly fluphenazine, haloperidol, and chlorpromazine, and other classes of psychotropic medicines. This within-typical antipsychotic switch might be due to limited access, availability, and unaffordability of atypical antipsychotics. A study done on schizophrenia clients’ treatment reported that 27.4% of antipsychotic treatment shifted. Among these, 47.4% treatment shift were due to the ineffectiveness of treatment [41].

In the current study, the percentage of clients who were prescribed two or more psychotropic medicines was 36.1%, which shows a polypharmacy practice. The current study result is higher than polypharmacy results reported from India (20%) and lower than studies reported from Amanuel Specialized Hospital (50%, polypharmacy) and Nekemte Referral Hospital (52.21%, polypharmacy) [17,21,42]. It is also lower than studies done by psychiatry hospitals in Hungary that showed 70% polypharmacy and in Nigeria that again reported 92.3% polypharmacy [18,30]. Polypharmacy leads to adverse drug effect burden, poor adherence, and unnecessary costs to service users, putting patients at risk of drug interactions [19]. The use of a combination of psychotropic medicines is reasonable in a few cases when justified by comorbidities, to manage medication side effects, and to enhance the efficacy of the first treatment. However, prescribing many psychotropic medicines is based on practical experience rather than evidence [43]. Hence, it is important to challenge polypharmacy prescribing malpractice by raising awareness among prescribers about the potential side effect burden. Providing targeted recommendations can help  prescribersto limit prescribing more than one psychotropic medicine unless clinically justified.

### Limitations of the study

Although a census approach was used to include all available eligible patients’ records during the study period, the study relied on paper-based medical records. Therefore, records that were lost, unavailable or incompletely documented could not be reviewed. This could introduce selection bias. Due to the nature of the cross-sectional study design, it was not possible to establish a cause-and-effect relationship. Additionally, given that SPH and HPH only began offering psychiatric outpatient services in 2017, the timing and availability of services could have influenced overall utilization figures.

## Conclusion

Typical antipsychotic medicines were the most commonly prescribed psychotropic medicines, followed by antidepressants and anticonvulsants/mood stabilizers. Chlorpromazine was the most frequently prescribed medicine from typical antipsychotics, followed by Risperidone from atypical. Amitriptyline was the most frequently prescribed antidepressant and Phenobarbitone was the most commonly utilized anticonvulsant. More than one-third of the psychiatric patients were prescribed psychotropic polypharmacy. Chlorpromazine and amitriptyline were the most combined psychotropic medicines, followed by haloperidol with amitriptyline and risperidone with fluoxetine. Treatment shifts occurred mostly due to poor improvement, relapse, side effects, and non-adherence. A further prospective continuous study that looks into both prevalence and consumption trends is needed to have more information on psychotropic utilisation patterns.

## Supporting information

S1 Data Deidentified data (SPSS). (SAV)

## Abbreviations

HPH: Hoomacho Primary Hospital
SPH: Shone Primary Hospital
SSRI: Selective Serotonin Reuptake Inhibitors
TCA: Tricyclic Antidepressants
WUNEMMCSH: Wachemo University Nigist Eleni Mohammed Memorial Comprehensive Specialized Hospital
