# Supplementary material for: Assessment of psychotropic medicines utilization patterns for the treatment of mental and neurological disorders in Public Hospitals of Hadiya Zone, Southern Ethiopia: A record-based study
Source: PLOS Ment Health. 2026 Aug 7;3(8):e0000665. doi: 10.1371/journal.pmen.0000665 (PMC13450774; doi:10.1371/journal.pmen.0000665)
Supplement: Supplementary file 1 [file pmen.0000665.s002.docx]

**Dear PLOS MENTAL HEALTH editor and reviewers!**

**Subject :** Rebuttal to decision and reviewers comments for manuscript ID **PONE-D-23-36615/Title-Assessment of psychotropic medicines utilization pattern in Hadiya Zone Public Hospitals, Southern Ethiopia.**

Thank you for your brief thoughtful review and the opportunity to respond to the comments and concerns raised by previous PLOS ONE reviewers. We appreciate the time and effort made in evaluating our work, and we have considered all comments given to prepare revised manuscript.

**Response to previous PLOS One reviewers**

We thank for your developmental comments provided by the reviewers. We have tried to consider all the developmental comments provided and have revised the manuscript accordingly to improve clarity, methodology and overall quality. We have provided a point by point response to the reviewer’s comments.

**Reviewer #1**

**Comment 1:** Abstract: Result: 11th line……The authors should revise the language to improve [readability.eg](http://readability.eg/). Among Antidepressants, Amitriptyline (TCA) was prescribed most frequent 74.9% followed by fluoxetine (SSRI) 21.8%.

**Response 1:** We thank the reviewer. We agree and have made the changes. We have revised the abstract to improve readability, especially in result section. Among antidepressants, amitriptyline was prescribed the most frequently, 74.9%, followed by fluoxetine, 21.8%.

**Comment 2:** Introduction: 1st paragraph: Second and third last lines: Repetition below
Statement of Problem: first paragraph,

**Response 2:** We thank the reviewer and removed repetitive sentences in the first paragraph.

**Comment 3**: second line: Mental, neurological, and substance use disorders (MNS), instead of Mental disorders

**Response 3:** Mental and neurologic disorders is correct terminology

**Comment 4:** Second para, last line: seems incomplete sentence

**Response 4:** Completed the incomplete sentences
**Methods and Materials:**

**Comment 5:** 14-15 line: Repetition

**Response 5:** Removed the repetitive sentences on lines 14-15
**Comment 6:** 16th line: who are primary mental health care providers in all hospitals, mention that 16th and 17th line: Clarify more data collection method especially: using medical charts review and using data abstraction format

**Response 6:** Clarified. Primary mental healthcare providers include psychiatrists and psychiatry nurses.

**Result part:**

**Comment 7:** Table 1, Note: Rewrite sentence and make it clear

**Response 7:** Rewritten table 1 note for clarity

**Comment 8:** 3rd paragraph: last sentence: Revise the language, red highlight: correct
Table 2: labelled as table 1: correct it,

**Response 8:** Corrected labelling to table 2

**Comment 9:** a. what diagnoses were included in PSYCHOSIS? b. why schizophrenia and Psychosis were kept in separate diagnosis entity?

**Response 9 :** Diagnosis included in psychosis only. Some cases were written relapsed psychosis and we took as written on patient charts. Schizophrenia and psychosis are categorized separately because they are two different mental disorders.
**Comment 10:** Para below table 2: 1st and 3rd line: grammatical mistakes
Para below table 3: WUNEMRTH were antipsychotics 793(51.2%) followed by …… Repetition of sentences. Table 4: labelled as 2
Table 6: The authors should clarify the following table OR revise it
Para 2nd, below table 7: 2-5th line: correct grammar,113% (36.8%): Do not start with number the beginning of sentence

**Response 10:** Corrected grammatical errors in the paragraph below table 2. Clarified the description of antipsychotics in table 3 and revised repetitions. Corrected the labeling of table 4 to table 2 and revised table 6 for clarity.

**Discussion:**
**Comment 11:** 2nd paragraph: 7th line: comment: lower or similar?
Last sentence: Most mental disorders onset……May not be suitable here, if possible rearrange else.
3rd paragraph: 2nd line: lower…..less or similar? than in Amanuel hospital
3rd line: community based…?

5-6th line: The discrepancy might be due…..rearrange and make it clear sentence
7th line: Awareness of society's depression ……….rearrange and make it clear sentence
4th paragraph: 9& 10th lines: shift of epilepsy…..rewrite and revise the language
12-13th line: This might be related to the availability….. details should be expanded and clarified to ensure that readers understand
21st line: Expanding…..please clarify this
5th paragraph: last two sentences…cost affordability……organize in a way so there is no repetition
6th paragraph
5-7 th lines….where SSRI represented (73%) of use whereas……Revise the language to improve readability eg: studies in different countries where SSRIs were prescribed more than TCA, Italy(... vs ...), France (.... vs ...)
8-10th lines: SSRIs are generally significantly……Rearrange: can be mentioned at first then arrange the findings.
Last line: psychotherapy……not appropriate word
7th para: 2nd line- %%....remove one
8th para: 2nd line..check spellings
7th line: psychosis depression……please put correct diagnostic term
9th para: 20th line: due to limited access…..revise the language eg. easy availability and affordability
25th line: study reported from India 2.1+0.8, please clarify this
31-33rd lines: polypharmacy ………….Remove unnecessary words
10th para: 2nd line….was 7.6 ?

11th para: 1st sentence: have shown majority of clients……Does Table 6 give this findings? if no, from where is it concluded?

4th line: WHO's recommended average consulting time……mention it
11-13th line: This was revealed in the fact ….. Revise the language to improve readability

**Response 11:** Rephrased sentences for clarity. Reorganized sentences to improve flow and clarity. Removed unnecessary words. Content was edited some sentences like average consulting time and knowledge of dispensed psychotropic were removed.

C**omment 12:** Limitation of study: not discussed

**Response 12:** We have included paragraph discussing the study limitations.

Conclusion and recommendation:

**Comment 13:** The authors should rewrite conclusion eg. pattern of mental disorder, mentioned in aims: not included
Chlorpromazine was the most ….revise the language
followed by Haloperidol with Amitriptyline …..remove unnecessary information
an increase in WUNEMRTH….??
knowledge of dispensed psychotropic……about what?

Response 13: We thank the reviewer and we have rewritten the conclusion. Our focus is psychotropic utilization pattern. To better describe the utilization pattern, we included the data of mental and neurologic disorders data. We have removed unnecessary sentences like knowledge of dispensed psychotropic were removed.

**Reviewer #2:**

We are grateful for the reviewer’s valuable feedback, which has significantly enhanced the overall quality and scholarly contribution of our work. We have seen your deep insight on the subject area and concern for development of study for publication. We have carefully revised the manuscript to address majority of comments and improve manuscript quality and overall clarity. Below, we provided a point-by-point response for the reviewers’ comment.
**Comment 1:** The authors start with the assumption that the psychotropic utilization pattern is irrational globally and in Ethiopia, both in abstract and in the introduction section. However, I do now see the conceptualization of irrational psychotropic use anywhere through the manuscript. Besides, it appears as a multicentre survey done at 3 hospitals. However, there is no information on how the patients visiting in the three areas differ in terms of their socio demographic profile.

**Response 1:** We acknowledge the concern regarding the conceptualization of irrational psychotropic medicine use. We have additionally clarified in introduction that our study aims to assess utilization patterns, which may include irrational prescribing, but primary focus is on describing the patterns rather than labeling the as irrational use.

**Comment 2:** There is no clear rationale doing the study.

**Response 2:** We thank the reviewer**.** We have further clarified the rational of study, emphasizing the importance of understanding utilization patterns in the context of Ethiopian mental health services and lack of recent data in study settings.

**Comment 3:** Another major flaw is with the methodology. There is no clarity on how the information was collected on the various factors and drug usage pattern. There is mention of usage of data abstraction format, prospective interview and observation, but again the manuscript falls short in elaborating the same. At times, it appears they did a medical chart review, at other times, it appears they did patient interview. It is tough to assess how the authors arrived at the conclusion of consultation time and prescribing times.
In psychiatry and mental health in general, the time for consultation is longer for the first-time visit compared to prospective visits and also depends on a host of factors, including possible diagnosis, insight level and so forth. There is no information on these as well. Besides, the basis of diagnosis is not clear on who made the diagnosis, if there was a standard format for diagnosis and classification was there a uniform protocol in all three sites assessed, what were the psychotropic available in all these sites etc. So, it is difficult to arrive at a conclusion based on available information. Further, the role of prescribers and dispensers is not clear, and it appears there is overlap of certain information.
Finally, the discussion does not adequately address the findings presented in light of the recent evidence. I therefore advise the authors to rewrite the manuscript considering all the points mentioned.
Response 3: Once again I thank you for the comments. We have made revision in entire content. First, we have limited our study to retrospective chart review only. Therefore, data was collected through retrospective chart review using prepared data collection tool. The same data abstraction format was used in all the three hospitals. Consultation time part is removed from current manuscript. Diagnosis made by psychiatrists and psychiatry nurses were taken directly from patient charts. We have revised discussion part to better address the findings in light of recent evidence. We have also included limitations of study.

We believe that these revisions have strengthened our manuscript. We kindly request you to consider our revised manuscript for publication.

Thank you again for your valuable feedback and opportunity to improve our work.

With regards,

Mengistu G.(Corresponding author)
