# Supplementary material for: Assessment of psychotropic medicines utilization patterns for the treatment of mental and neurological disorders in Public Hospitals of Hadiya Zone, Southern Ethiopia: A record-based study
Source: PLOS Ment Health. 2026 Aug 7;3(8):e0000665. doi: 10.1371/journal.pmen.0000665 (PMC13450774; doi:10.1371/journal.pmen.0000665)
Supplement: Supplementary file 2 [file pmen.0000665.s003.docx]

**Dear PLOS MENTAL HEALTH editor and reviewers!**

**Subject:** Response for reviewers’ developmental comments for manuscript [PMEN-D-26-00125] with Title-Assessment of psychotropic medicines utilization pattern in Hadiya Zone Public Hospitals, Southern Ethiopia.

**Dear reviewers!**

Thank you for your detailed and developmental comments. Please find below our response and necessary amendments. We appreciate the time and effort made in evaluating our work, and we have considered all comments given to prepare revised manuscript.

**Response to PLOS Mental reviewers**

**Reviewer #1**

**Comment 1:** AUTHORSHIP DISCREPANCY

The third author is listed as "Alemayehu Adnew" in the manuscript metadata but as

"Alemayehu Teshale" with a different email address in the manuscript body. These are two distinct names, not typographical variants. The authors must clarify the correct identity of the third author and ensure consistency across all submission records. The PLOS authorship integrity policy requires this to be resolved transparently

**Response 1:** We thank you for pointing out the inconsistency regarding the third author’s name. The correct name is "Alemayehu Adnew Teshale". He was my co-advisor. We have now revised all submission files to ensure consistent author information thought.

**Comment 2:** CRITICAL PHARMACOLOGICAL ERROR IN THE CONCLUSION

The Conclusion states: "Chlorpromazine was the most frequently prescribed medicine from atypical antipsychotics.” This is factually incorrect. Chlorpromazine is a first-generation (typical/conventional) antipsychotic — not an atypical one. The only atypical antipsychotic reported in this study is risperidone. This error must be corrected throughout, as it directly contradicts the Results and undermines the clinical credibility of the manuscript.

**Response 2:** We apologize the error regarding chlorpromazine. We have corrected the manuscript to state chlorpromazine is first generation antipsychotic.

**Comment 3**: FLUOXETINE MISCLASSIFIED AS A TRICYCLIC ANTIDEPRESSANT

Both the Abstract and main text state: "Among tricyclic antidepressants, amitriptyline

was prescribed most frequently, 74.9%, followed by fluoxetine, 21.8%. "Fluoxetine is

a selective serotonin reuptake inhibitor (SSRI), not a tricyclic antidepressant (TCA).

This is a fundamental pharmacological error.

**Response 3:** We again apologize the error regarding fluoxetine. As you recommended, the sentence was revised: "Among antidepressants, amitriptyline (TCA) was most frequently prescribed (74.9%), followed by fluoxetine (SSRI) (21.5%)."

**Comment 4:** NUMERICAL INCONSISTENCY: TREATMENT SHIFT RATE. The Abstract reports a treatment shift rate of 12.33%; the Results section calculates 139/1200 = 11.58%; the discussion refers to 11.5%. Three different values are used for the same outcome. The authors must verify the correct figure and apply it consistently throughout.

**Response 4:** We thank you for pointing out the inconsistency regarding numeric inconsistency. We reviewed all calculations and confirmed the correct treatment shift rate is 39/1200 = 11.58%. We have updated the abstract, results and discussions to reflect this consistency.

**Comment 5:** 5. SPSS VERSION CONFLICT. The Abstract states analyses were performed with "SPSS version 20"; the Methods section states "SPSS version 26." These are different software versions. The correct version must be confirmed and stated consistently.

**Response 5:** Thank again for pointing out inconsistency. The statistical analyses were conducted using SPSS version 26.
**Comment 6:** ANTIPSYCHOTIC ARITHMETIC ERROR

The Discussion states typical antipsychotics were prescribed in 616/835 cases (73.86%)

and atypical antipsychotics in 216/835 (26.14%). However, 616 + 216 = 832, not 835.

Furthermore, Table 5 reports total antipsychotics as 834, not 835. All figures must be

verified and reconciled. The disposition of any cases not counted in either subgroup

(e.g., injectable haloperidol, olanzapine, fluphenazine) must be explicitly addressed.

**Response 6:** We thank you for pointing out the inconsistency regarding numeric inconsistency. We reviewed all calculations and confirmed the correct total of ANTIPSYCHOTIC is 834. Olanzapine was prescribed for two patients (table 5). 616 + 218 = 834. We have updated discussions to reflect this consistency.

**Comment 7:** THIORIDAZINE: UNADDRESSED PATIENT SAFETY CONCERN
Thioridazine constituted 4.3% of all psychotropic prescriptions (74/1734) and was
among the three most used antipsychotics. Thioridazine was withdrawn from most markets
in the early 2000s due to dose-dependent QTc prolongation and risk of fatal ventricular
arrhythmias (torsades de pointes). The manuscript makes no mention of this regulation.
history, does not discuss cardiac safety implications, and does not describe whether
any monitoring was in place. This omission has material patient safety implications.
At minimum, the authors must: (a) describe the regulatory status of thioridazine in
Ethiopia during the study period; (b) discuss cardiac monitoring availability; and
(c) frame this finding in terms of Pharmacovigilance policy for resource-limited settings.

**Response 7:** At the time of this study, due to limited availability and accessibility of psychotropic medicines especially in remote area health facilities, thioridazine was among the commonly prescribed psychotropic medicines. Thioridazine has been significantly linked to aberrant ECG readings. In Ethiopia, thioridazine was used under specialist supervision, as second line treatment of schizophrenia in adults. The patients were put on follow up by ECG screening and electrolyte measurement before treatment, after each dose increase and at 6 month intervals. Besides, the patients were monitored for visual defects on prolonged use. Antipsychotic, for example, thioridazine has been significantly linked to aberrant ECG readings. Currently, availability and accessibility of psychotropic medicines especially second generation antipsychotics have improved and use of thioridazine is minimal.

**Comment 8:** DATA AVAILABILITY DOES NOT MEET PLOS POLICY

The Data Availability Statement reads: "datasets will be available from the corresponding author on reasonable request." PLOS requires that all underlying data be made freely available without restriction — not merely on request. The authors must deposit a de-identified dataset in a public repository (e.g., Zenodo, OSF, Figshare) and provide a persistent identifier, or provide a specific, documented legal/ethical justification for restriction. This is a journal compliance issue that will prevent acceptance if unresolved.

**Response 8:** All relevant data are within the manuscript and its Supporting Information files.

**Comment 9:** DIAGNOSTIC CRITERIA NOT SPECIFIED. The manuscript does not state which diagnostic classification system (ICD-10 or DSM-5) was used, whether diagnoses were clinician-assigned using a validated framework, or whether diagnostic practice was consistent across three hospitals with very different staffing structures. This must be clarified, as it affects the reproducibility and interpretability of all prevalence figures.

**Response 9:** The diagnosis is based on Diagnostic and Statistical Manual of Mental Disorders (DSM)-5 (American Psychiatric Association, 2013) assigned by clinicians at each hospital. The diagnostic practice was consistent across three hospitals.

**Comment 10:** PSYCHOSIS vs. SCHIZOPHRENIA: CONCEPTUAL OVERLAP
Schizophrenia is a subtype of psychosis. Reporting them as independent diagnostic
categories (27.5% psychosis; 15.67% schizophrenia) is clinically problematic unless
"Psychosis" is explicitly defined as non-schizophrenia psychosis. Similarly, the
"relapsed psychosis" category (n=61) must be clarified as to whether it is drawn from
within the psychosis total or is a separate group. The authors must define all
diagnostic categories operationally.

**Response 10:** Thank you for your feedback. In our study, we utilized diagnosis written on patients charts assigned by psychiatry professionals during routine clinical assessment. These diagnoses reflect real world clinical practice and based on established diagnostic criteria, as per the assessment protocols used in the study facilities. Our data collection focused on recorded diagnoses to accurately reflect the clinical diagnoses made in practice, rather than re-evaluating or redefining the diagnoses retrospectively. Psychosis and Schizophrenia are written separately. I think it is appropriate to identify between psychosis and schizophrenia for better management of patient. Psychosis is a symptom of detachment from reality, such as hallucinations or delusions. It became Schizophrenia when symptoms persist continuously for more than six months and are accompanied by significant decline in daily functioning. Some cases were written relapsed psychosis and we took as written on patient charts.

**Comment 11:** PRESCRIBER HETEROGENEITY INADEQUATELY ADDRESSED
WUNEMRH is staffed by psychiatrists; SPH and HPH have only psychiatry nurses, and
opened psychiatric OPD services only in 2017—mid-study. Pooled aggregate data across
These three very different settings obscure important variation. The manuscript must
more explicitly discuss how this structural heterogeneity affects the interpretation
of aggregated utilization figures in Tables 4 and 5.

**Response 11:** Thank for indicating the issue of prescribers heterogeneity across the study sites while WUNEMRH is staffed by psychiatrists and SPH and HPH are primary staffed by psychiatry nurses with psychiatric outpatient services only starting in 2027. We believe that this structural heterogeneity does not significantly affect the interpretation of aggregate utilization figures presented in table 4 and 5. The findings are displayed separately into referral hospital and primary hospitals results.

This is because the medication utilization data are based on the number of patients served at each facility, and the core service provision- focused on psychiatric outpatient care is similar across all sites. Due to limited access for mental healthcare service in developing countries, primary healthcare facilities were considered to provide mental healthcare service. Primary healthcare facilities are located at remote areas. Due to this, the number of service utilizers and access for psychotropic medicines could be limited. But the prescribing practice, guided by protocols and clinical guidelines, are consistent irrespective of staff composition. Due to limited psychiatry professionals, psychiatry nurses were assigned to take the responsibility psychiatry service both in primary and referral hospitals. In our study, WUNEMRH had also psychiatry nurses.

Nonetheless, we acknowledge this as limitation and we will include in discussion of how this structural differences may influence the findings in the revised manuscript.

C**omment 12:** TABLE 6 IS UNINTERPRETABLE. The co-prescribing matrix (Table 6) appears to show individual drug totals on the
diagonal (e.g., Chlorpromazine=259, Amitriptyline=334), which are not co-prescription
counts. Off-diagonal cells appear to represent combination counts, but this is not
stated. The table must be accompanied by a clear explanatory footnote defining what
each cell represents it, and the diagonal values must be distinguished from combination data.

**Response 12:** The footnote explaining the structure of the co prescribing matrix, including clarification of diagonal values as individual drugs totals has been added.

**Comment 13:** POLYPHARMACY DEFINITION IS NON-STANDARD
The study defines polypharmacy as receiving two or more psychotropic medicines. The
The standard clinical definition of polypharmacy is five or more concurrent medications.
While "psychotropic polypharmacy" at a ≥2 threshold is used in some psychiatric
literature, the authors must explicitly define their threshold upfront and note that
it may inflate the reported polypharmacy rate relative to studies using stricter
definitions — especially when making cross-country comparisons in the Discussion.

**Response 13:** Thank you for concern. The manuscript now explicitly defines polypharmacy at the ≥ 2 psychotropic medicines threshold and discusses how this differs from the standard clinical definition, nothing implications for comparisons.

**Comment 14:** WHO/INRUD INDICATORS NOT FORMALLY APPLIED
Drug utilization studies achieve greatest international comparability when they employ
WHO/INRUD core prescribing indicators. This study partially captures relevant data
(average medicines per encounter = 1.5; injectable rate = 7.6%) but does not frame
them within the WHO/INRUD framework. The authors should either formally present
applicable WHO/INRUD indicators or clearly state why this framework was not used.

**Response 14:** We appreciate your suggestion. While the WHO/INRUD core prescribing indicators are widely used for assessing rational use and facilitating international comparisons, the present study was not designed a formal WHO/INRUD core prescribing indicators evaluation. The available dataset permitted calculations of only selected prescribing measures, such as average number of medicines per encounter and the proportion of encounters involving injectable medicines.

**Comment 15:** TEMPORAL TRENDS ARE CONFOUNDED
SPH and HPH only began psychiatric services in 2017. Any temporal trend presented
across all five years for the pooled dataset is confounded: changes in the aggregate
numbers reflect service expansion, not genuine shifts in prescribing practice. The
Discussion should clearly acknowledge this structural limitation and avoid
over-interpreting pooled trend data.

**Response 15:** This has been addresses in response 11.

**Comment 16:** CENSUS APPROACH NOT FORMALLY STATED
The authors state, "all eligible records were included" due to small patient numbers.
But frame this as a sample ("Sample sizes of 1200 charts were included"). The authors
should explicitly describe this as a census approach, report the total number of
records reviewed vs. excluded, specify the exclusion criteria applied, and discuss
potential selection bias from paper-based chart loss.

**Response 16:** We expanded the limitations section to address key issues such as retrospective data accuracy and cautious interpretation of aggregated data.

**Comment 17:** LIMITATIONS SECTION IS INSUFFICIENT
Despite being added following prior review, the limitations discussion remains thin.
Key limitations that must be discussed include (a) retrospective chart review is
vulnerable to documentation errors and missing data; (b) the study captures
prescriptions, not dispensing or actual medicine use; (c) diagnoses were taken
from charts without validation or inter-rater reliability assessment; (d) the
A cross-sectional design precludes causal inference about prescribing determinants.

**Response 17:** We expanded the limitations section to address key issues such as retrospective data accuracy, cross sectional study design limitation and cautious interpretation of aggregated data.

**Comment 18:** Table 2 contains a typographical error: "from 86.5% in 2015 to 16.7% in 2011 in SPH." should read "2019."

**Response 18:** Thank for your comment. It is edited.

**Comment 19:** The hospital abbreviation is used inconsistently: "WUNEMH," "WUNEMRTH," and "WUNEMRH"
all appear. One abbreviation must be chosen and used consistently throughout, including
in the Abbreviations list.

**Response 19:** Corrected

**Comment 20:** The Discussion states amitriptyline = 334/446 (78.5%). The correct calculation is 334/446 = 74.9%. This arithmetic error must be corrected.

**Response 20:** Corrected

**Comment 21:** The abstract states fluoxetine is 21.8% of antidepressants. The correct calculation from Table 5 is 96/446 = 21.5%. Please verify and correct.

**Response 21:** Corrected

**Comment 22:** The Discussion refers to SSRIs as "first-line psychotherapy for depression." The correct term is "pharmacotherapy." Psychotherapy refers to talking therapies (e.g.,
CBT). This error was flagged by a prior reviewer and remains uncorrected.

**Response 22:** Corrected
**Comment 23:** Figure 1 does not appear in the reviewer PDF. If this is a submission formatting issue, figures must be embedded in the revised manuscript.
**Response 23:** Corrected
**Comment 24:**. The rebuttal letter references a prior submission to PLOS ONE (PONE-D-23-36615).The editorial office should confirm whether this transfer has been formally disclosed
as part of submission.

**Response 24:** it has been formally disclosed per editorial requirement.

**Reviewer #2:**  We are grateful for the reviewer’s valuable feedback, which has significantly enhanced the overall quality and scholarly contribution of our work. We have seen your deep insight on the subject area and concern for development of study for publication. We have carefully revised the manuscript to address majority of comments and improve manuscript quality and overall clarity. Below, we provided a point-by-point response for the reviewer.
**Comment 1:** You talk about mental and neurologic disorders, it is better to specify what are the mental disorders and neurologic disorders in your findings.
The methodology and results need readjusted referring to these comments below:

**Response 1:** We thank you for this valuable comment. We agree that the distinction between mental and neurologic disorders should be clearly specified. We have revised manuscript to clearly specify the two categories in method and result sections.

**Comment 2:** What was your study period (time of collecting data, given that you obtained the ethical approval in 2019). Your study was retrospective. How have you interacted with the patients to know the various factors you have mentioned?

**Response 2:** The study period for data collection was from June 15 December 30 2019 immediately after ethical approval. This study was conducted as retrospective chart review and there was no direct interaction with patients and patients were not available at that time in the hospitals. Charts were reviewed after getting ethical approval and permission from hospital administration.

**Comment 3:** What is the difference between Schizophrenia and psychosis. Isn't schizophrenia one of the groups of psychosis? You need to use accepted diagnosis in ICD 10 or DSM-5 specify the tools used to get the diagnosis
Is a seizure a diagnosis or a symptom of another mental or neurologic disorder? Use acceptable diagnosis in known international classifications.
Or if your objectives were not mainly based on diagnosis, specify it.
**Response 3:** Once again I thank you for the comments. The diagnosis is based on Diagnostic and Statistical Manual of Mental Disorders (DSM)-5 (American Psychiatric Association, 2013) assigned by clinicians at each hospital. In our study, we utilized diagnosis written on patients charts assigned by psychiatry professionals during routine clinical assessment. These diagnoses reflect real world clinical practice and based on established diagnostic criteria, as per the assessment protocols used in the study facilities. Our data collection focused on recorded diagnoses to accurately reflect the clinical diagnoses made in practice, rather than re-evaluating or redefining the diagnoses retrospectively. Psychosis and Schizophrenia are written separately. I think it is appropriate to identify between psychosis and schizophrenia for better management of patient. Psychosis is a symptom of detachment from reality, such as hallucinations or delusions. It became Schizophrenia when symptoms persist continuously for more than six months and are accompanied by significant decline in daily functioning. As you know well seizure is sudden surge of uncontrolled electrical activity in the brain which causes temporary changes in movement, behavior awareness. Epilepsy is a chronic neurologic disorder diagnosed when someone has two or more unprovoked seizures. I think it is appropriate to write separately type of disorder for better management purpose.

**Comment 4.** Specify why data are available at reasonable request from the author according to PLOS policy (ethical concern? protection of patient's information...

**Response 4:** We updated the data availability statement to comply with fully with PLOS policy providing data in manuscript as supplementary file. Ethical considerations regarding patient privacy have been addressed by removing personal identifiers.

We believe that these revisions have strengthened our manuscript. We kindly request you to consider our revised manuscript for publication.

Thank you again for your valuable feedback and offering opportunity to improve our work.

With regards,

Mengistu G.(Corresponding author)
