# Supplementary material for: Assessment of psychotropic medicines utilization patterns for the treatment of mental and neurological disorders in Public Hospitals of Hadiya Zone, Southern Ethiopia: A record-based study
Source: PLOS Ment Health. 2026 Aug 7;3(8):e0000665. doi: 10.1371/journal.pmen.0000665 (PMC13450774; doi:10.1371/journal.pmen.0000665)
Supplement: Peer review history

# Author comment: Author response to Decision Letter 0

**DOI:** 10.1371/journal.pmen.0000665.r001

**Transfer Alert**

This paper was transferred from another journal. As a result, its full editorial history (including decision letters, peer reviews and author responses) may not be present.

21 Mar 2026

Attachment Submitted filename: Response to Reviewers.docx

# Peer review: Decision Letter 0

**DOI:** 10.1371/journal.pmen.0000665.r002

**Authors:** Karli Montague-Cardoso

2 Jun 2026

PMEN-D-26-00125

Assessment of Psychotropic Medicines Utilization Pattern for Treatment of Mental and Neurological Disorders in Hadiya Zone Public Hospitals, Southern, Ethiopia

PLOS Mental Health

Dear Dr. Tirore,

Thank you for submitting your manuscript to PLOS Mental Health. After careful consideration, we feel that it has merit but does not fully meet PLOS Mental Health’s publication criteria as it currently stands. Therefore, we invite you to submit a revised version of the manuscript that addresses the points raised during the review process. Please note that all comments, which you can find below, must be fully addressed in order for us to proceed with the manuscript. With respect to the comments from the reviewers regarding data availability, if you are unable to provide raw, deidentified data, please provide a detailed explanation regarding this.

Please submit your revised manuscript by Jul 30 2026 11:59PM. If you will need more time than this to complete your revisions, please reply to this message or contact the journal office at mentalhealth@plos.org. When you're ready to submit your revision, log on to https://www.editorialmanager.com/pmen/ and select the 'Submissions Needing Revision' folder to locate your manuscript file.

Please include the following items when submitting your revised manuscript:

- A letter that responds to each point raised by the editor and reviewer(s). You should upload this letter as a separate file labeled 'Response to Reviewers'.
- A marked-up copy of your manuscript that highlights changes made to the original version. You should upload this as a separate file labeled 'Revised Manuscript with Track Changes'.
- An unmarked version of your revised paper without tracked changes. You should upload this as a separate file labeled 'Manuscript'.

Guidelines for resubmitting your figure files are available below the reviewer comments at the end of this letter.

As the corresponding author, your ORCID iD is verified in the submission system and will appear in the published article. PLOS supports the use of ORCID, and we encourage all coauthors to register for an ORCID iD and use it as well. Please encourage your coauthors to verify their ORCID iD within the submission system before final acceptance, as unverified ORCID iDs will not appear in the published article. *Only*  the individual author can complete the verification step; PLOS staff *cannot*  verify ORCID iDs on behalf of authors.

We look forward to receiving your revised manuscript.

Kind regards,

Karli Montague-Cardoso

Staff Editor

PLOS Mental Health

Journal Requirements:

1. Please ensure that your Ethics Statement is available in its entirety at the beginning of your Methods section, under a subheading 'Ethics Statement'.

2. Please amend your detailed Financial Disclosure statement. This is published with the article. It must therefore be completed in full sentences and contain the exact wording you wish to be published.

a. Please clarify all sources of funding (financial or material support) for your study. List the grants (with grant number) or organizations (with url) that supported your study, including funding received from your institution.

b. State the initials, alongside each funding source, of each author to receive each grant.

c. State what role the funders took in the study. If the funders had no role in your study, please state: “The funders had no role in study design, data collection and analysis, decision to publish, or preparation of the manuscript.”

d. If any authors received a salary from any of your funders, please state which authors and which funders.

3. In the online submission form, you indicated that "The datasets used and/or analyzed during the current study will be available from the corresponding author on reasonable request.".

All PLOS journals now require all data underlying the findings described in their manuscript to be freely available to other researchers, either

1. In a public repository,

2. Within the manuscript itself, or

3. Uploaded as supplementary information.

This policy applies to all data except where public deposition would breach compliance with the protocol approved by your research ethics board. If your data cannot be made publicly available for ethical or legal reasons (e.g., public availability would compromise patient privacy), please explain your reasons by return email and your exemption request will be escalated to the editor for approval. Your exemption request will be handled independently and will not hold up the peer review process, but will need to be resolved should your manuscript be accepted for publication. One of the Editorial team will then be in touch if there are any issues.

If the reviewer comments include a recommendation to cite specific previously published works, please review and evaluate these publications to determine whether they are relevant and should be cited. There is no requirement to cite these works unless the editor has indicated otherwise.

Additional Editor Comments (if provided):

[Note: HTML markup is below. Please do not edit.]

Reviewers' comments:

Reviewer's Responses to Questions

**Comments to the Author**

1. Does this manuscript meet PLOS Mental Health’s publication criteria? Is the manuscript technically sound, and do the data support the conclusions? The manuscript must describe methodologically and ethically rigorous research with conclusions that are appropriately drawn based on the data presented.

Reviewer #1: No

Reviewer #2: Partly

**********

2. Has the statistical analysis been performed appropriately and rigorously?

Reviewer #1: No

Reviewer #2: Yes

**********

3. Have the authors made all data underlying the findings in their manuscript fully available (please refer to the Data Availability Statement at the start of the manuscript PDF file)?

The PLOS Data policy requires authors to make all data underlying the findings described in their manuscript fully available without restriction, with rare exception. The data should be provided as part of the manuscript or its supporting information, or deposited to a public repository. For example, in addition to summary statistics, the data points behind means, medians and variance measures should be available. If there are restrictions on publicly sharing data—e.g. participant privacy or use of data from a third party—those must be specified.

Reviewer #1: No

Reviewer #2: Yes

**********

4. Is the manuscript presented in an intelligible fashion and written in standard English?

PLOS Mental Health does not copyedit accepted manuscripts, so the language in submitted articles must be clear, correct, and unambiguous. Any typographical or grammatical errors should be corrected at revision, so please note any specific errors here.

Reviewer #1: No

Reviewer #2: Yes

**********

5. Review Comments to the Author

Please use the space provided to explain your answers to the questions above. You may also include additional comments for the author, including concerns about dual publication, research ethics, or publication ethics. (Please upload your review as an attachment if it exceeds 20,000 characters)

Reviewer #1: RECOMMENDATION: Major Revision

This manuscript reports a retrospective, facility-based cross-sectional study of

psychotropic medicine utilization across three public hospitals in Hadiya Zone,

Ethiopia (2015–2019; n=1,200 charts). The topic is relevant and addresses a genuine

evidence gap in psychopharmacoepidemiology for low-income settings. However, the

manuscript contains multiple critical errors—including pharmacological

misclassification of drugs, numerical inconsistencies across sections, authorship

discrepancies, and data availability non-compliance—that require full correction

before publication. I recommend major revision.

=== MAJOR CONCERNS ===

1. AUTHORSHIP DISCREPANCY

The third author is listed as "Alemayehu Adnew" in the manuscript metadata but as

"Alemayehu Teshale" with a different email address in the manuscript body. These are

two distinct names, not typographical variants. The authors must clarify the correct

identity of the third author and ensure consistency across all submission records.

The PLOS authorship integrity policy requires this to be resolved transparently.

2. CRITICAL PHARMACOLOGICAL ERROR IN THE CONCLUSION

The Conclusion states: "Chlorpromazine was the most frequently prescribed medicine

from atypical antipsychotics. "This is factually incorrect. Chlorpromazine is a

first-generation (typical/conventional) antipsychotic — not an atypical one. The

The only atypical antipsychotic reported in this study is risperidone. This error must

be corrected throughout, as it directly contradicts the Results and undermines the

clinical credibility of the manuscript.

3. FLUOXETINE MISCLASSIFIED AS A TRICYCLIC ANTIDEPRESSANT

Both the Abstract and main text state: "Among tricyclic antidepressants, amitriptyline

was prescribed most frequently, 74.9%, followed by fluoxetine, 21.8%. "Fluoxetine is

a selective serotonin reuptake inhibitor (SSRI), not a tricyclic antidepressant (TCA).

This is a fundamental pharmacological error. The sentence must be revised—for

example: "Among antidepressants, amitriptyline (TCA) was most frequently prescribed

(74.9%), followed by fluoxetine (SSRI) (21.8%)."

4. NUMERICAL INCONSISTENCY: TREATMENT SHIFT RATE

The Abstract reports a treatment shift rate of 12.33%; the Results section calculates

139/1200 = 11.58%; the discussion refers to 11.5%. Three different values are used

for the same outcome. The authors must verify the correct figure and apply it

consistently throughout.

5. SPSS VERSION CONFLICT

The Abstract states analyses were performed with "SPSS version 20"; the Methods section

states "SPSS version 26." These are different software versions. The correct version

must be confirmed and stated consistently.

6. ANTIPSYCHOTIC ARITHMETIC ERROR

The Discussion states typical antipsychotics were prescribed in 616/835 cases (73.86%)

and atypical antipsychotics in 216/835 (26.14%). However, 616 + 216 = 832, not 835.

Furthermore, Table 5 reports total antipsychotics as 834, not 835. All figures must be

verified and reconciled. The disposition of any cases not counted in either subgroup

(e.g., injectable haloperidol, olanzapine, fluphenazine) must be explicitly addressed.

7. THIORIDAZINE: UNADDRESSED PATIENT SAFETY CONCERN

Thioridazine constituted 4.3% of all psychotropic prescriptions (74/1734) and was

among the three most used antipsychotics. Thioridazine was withdrawn from most markets

in the early 2000s due to dose-dependent QTc prolongation and risk of fatal ventricular

arrhythmias (torsades de pointes). The manuscript makes no mention of this regulation.

history, does not discuss cardiac safety implications, and does not describe whether

any monitoring was in place. This omission has material patient safety implications.

At minimum, the authors must: (a) describe the regulatory status of thioridazine in

Ethiopia during the study period; (b) discuss cardiac monitoring availability; and

(c) frame this finding in terms of pharmacovigilance policy for resource-limited settings.

8. DATA AVAILABILITY DOES NOT MEET PLOS POLICY

The Data Availability Statement reads: "datasets will be available from the corresponding

author on reasonable request." PLOS requires that all underlying data be made freely

available without restriction — not merely on request. The authors must deposit a

de-identified dataset in a public repository (e.g., Zenodo, OSF, Figshare) and provide

a persistent identifier, or provide a specific, documented legal/ethical justification

for restriction. This is a journal compliance issue that will prevent acceptance if

unresolved.

9. DIAGNOSTIC CRITERIA NOT SPECIFIED

The manuscript does not state which diagnostic classification system (ICD-10 or DSM-5)

was used, whether diagnoses were clinician-assigned using a validated framework, or

whether diagnostic practice was consistent across three hospitals with very different

staffing structures. This must be clarified, as it affects the reproducibility and

interpretability of all prevalence figures.

10. PSYCHOSIS vs. SCHIZOPHRENIA: CONCEPTUAL OVERLAP

Schizophrenia is a subtype of psychosis. Reporting them as independent diagnostic

categories (27.5% psychosis; 15.67% schizophrenia) is clinically problematic unless

"Psychosis" is explicitly defined as non-schizophrenia psychosis. Similarly, the

"relapsed psychosis" category (n=61) must be clarified as to whether it is drawn from

within the psychosis total or is a separate group. The authors must define all

diagnostic categories operationally.

11. PRESCRIBER HETEROGENEITY INADEQUATELY ADDRESSED

WUNEMRH is staffed by psychiatrists; SPH and HPH have only psychiatry nurses, and

opened psychiatric OPD services only in 2017—mid-study. Pooled aggregate data across

These three very different settings obscure important variation. The manuscript must

more explicitly discuss how this structural heterogeneity affects the interpretation

of aggregated utilization figures in Tables 4 and 5.

=== MODERATE CONCERNS ===

12. TABLE 6 IS UNINTERPRETABLE

The co-prescribing matrix (Table 6) appears to show individual drug totals on the

diagonal (e.g., Chlorpromazine=259, Amitriptyline=334), which are not co-prescription

counts. Off-diagonal cells appear to represent combination counts, but this is not

stated. The table must be accompanied by a clear explanatory footnote defining what

each cell represents it, and the diagonal values must be distinguished from the combination.

data.

13. POLYPHARMACY DEFINITION IS NON-STANDARD

The study defines polypharmacy as receiving two or more psychotropic medicines. The

The standard clinical definition of polypharmacy is five or more concurrent medications.

While "psychotropic polypharmacy" at a ≥2 threshold is used in some psychiatric

literature, the authors must explicitly define their threshold upfront and note that

it may inflate the reported polypharmacy rate relative to studies using stricter

definitions — especially when making cross-country comparisons in the Discussion.

14. WHO/INRUD INDICATORS NOT FORMALLY APPLIED

Drug utilization studies achieve greatest international comparability when they employ

WHO/INRUD core prescribing indicators. This study partially captures relevant data

(average medicines per encounter = 1.5; injectable rate = 7.6%) but does not frame

them within the WHO/INRUD framework. The authors should either formally present

applicable WHO/INRUD indicators or clearly state why this framework was not used.

15. TEMPORAL TRENDS ARE CONFOUNDED

SPH and HPH only began psychiatric services in 2017. Any temporal trend presented

across all five years for the pooled dataset is confounded: changes in the aggregate

numbers reflect service expansion, not genuine shifts in prescribing practice. The

Discussion should clearly acknowledge this structural limitation and avoid

over-interpreting pooled trend data.

16. CENSUS APPROACH NOT FORMALLY STATED

The authors state, "all eligible records were included" due to small patient numbers.

But frame this as a sample ("Sample sizes of 1200 charts were included"). The authors

should explicitly describe this as a census approach, report the total number of

records reviewed vs. excluded, specify the exclusion criteria applied, and discuss

potential selection bias from paper-based chart loss.

17. LIMITATIONS SECTION IS INSUFFICIENT

Despite being added following prior review, the limitations discussion remains thin.

Key limitations that must be discussed include (a) retrospective chart review is

vulnerable to documentation errors and missing data; (b) the study captures

prescriptions, not dispensing or actual medicine use; (c) diagnoses were taken

from charts without validation or inter-rater reliability assessment; (d) the

A cross-sectional design precludes causal inference about prescribing determinants.

=== MINOR CONCERNS ===

18. Table 2 contains a typographical error: "from 86.5% in 2015 to 16.7% in 2011 in SPH."

should read "2019."

19. The hospital abbreviation is used inconsistently: "WUNEMH," "WUNEMRTH," and "WUNEMRH"

all appear. One abbreviation must be chosen and used consistently throughout, including

in the Abbreviations list.

20. The Discussion states amitriptyline = 334/446 (78.5%). The correct calculation is

334/446 = 74.9%. This arithmetic error must be corrected.

21. The abstract states fluoxetine is 21.8% of antidepressants. The correct calculation

from Table 5 is 96/446 = 21.5%. Please verify and correct.

22. The Discussion refers to SSRIs as "first-line psychotherapy for depression." The

The correct term is "pharmacotherapy." Psychotherapy refers to talking therapies (e.g.,

CBT). This error was flagged by a prior reviewer and remains uncorrected.

23. Figure 1 does not appear in the reviewer PDF. If this is a submission formatting

issue, figures must be embedded in the revised manuscript.

24. The rebuttal letter references a prior submission to PLOS ONE (PONE-D-23-36615).

The editorial office should confirm whether this transfer has been formally disclosed

as part of submission.

=== QUESTIONS FOR AUTHORS ===

(a) What is the correct full name of the third author?

(b) Which SPSS version was used — 20 or 26?

(c) What is the correct treatment shift rate—12.33%, 11.58%, or 11.5%?

(d) What was thioridazine's regulatory status in Ethiopia during 2015–2019? and

What cardiac safety monitoring was in place?

(e) In Table 6, do the diagonal values represent individual drug totals? If so,

Please add a footnote clarifying the structure of the matrix.

(f) What is the specific legal or ethical barrier to full open data deposit?

Reviewer #2: 1. You talk about mental and neurologic disorders, it is better to specify what are the mental disorders and neurologic disorders in your findings.

The methodology and results need reajusted refering to these comments below:

2.What was your study period (time of collecting data, given that you obtained the ethical approval in 2019)

Your study was retrospective. How have you interacted with the patients to know the various factors you have mentionned?

3. What is the difference between Schizophrenia and psychosis. Isn't schizophrenia one of the group of psychosis. You need to use accepted diagnosis in ICD 10 or DSM-5

specify the tools used to get the diagnosis

Is a seizure a diagnosis or a symptom of another mental or neurologic disorder? Use acceptable diagnosis in known international classifications.

Or if your objectives were not mainly based on diagnosis, specify it

4. Specify why data are avilable at reasonnable request from the author according to plos policy ( ethichal concern?, protection of patient's information?,....

**********

6. PLOS authors have the option to publish the peer review history of their article (what does this mean?). If published, this will include your full peer review and any attached files.

**Do you want your identity to be public for this peer review?**  If you choose “no”, your identity will remain anonymous but your review may still be made public.

For information about this choice, including consent withdrawal, please see our Privacy Policy.

Reviewer #1: **Yes:** Mohamed Boulahia

Reviewer #2: No

**********

[NOTE: If reviewer comments were submitted as an attachment file, they will be attached to this email and accessible via the submission site. Please log into your account, locate the manuscript record, and check for the action link "View Attachments". If this link does not appear, there are no attachment files.]

-->Figure Resubmissions:

While revising your submission, we strongly recommend that you use PLOS’s NAAS tool (https://ngplosjournals.pagemajik.ai/artanalysis) to test your figure files. NAAS can convert your figure files to the TIFF file type and meet basic requirements (such as print size, resolution), or provide you with a report on issues that do not meet our requirements and that NAAS cannot fix.

After uploading your figures to PLOS’s NAAS tool - https://ngplosjournals.pagemajik.ai/artanalysis, NAAS will process the files provided and display the results in the "Uploaded Files" section of the page as the processing is complete. If the uploaded figures meet our requirements (or NAAS is able to fix the files to meet our requirements), the figure will be marked as "fixed" above. If NAAS is unable to fix the files, a red "failed" label will appear above. When NAAS has confirmed that the figure files meet our requirements, please download the file via the download option, and include these NAAS processed figure files when submitting your revised manuscript.

# Author comment: Author response to Decision Letter 1

**DOI:** 10.1371/journal.pmen.0000665.r003

20 Jun 2026

Attachment Submitted filename: Response to Reviewer.docx

# Editor report: Decision Letter 1

**DOI:** 10.1371/journal.pmen.0000665.r004

**Authors:** Karli Montague-Cardoso

7 Jul 2026

Assessment of Psychotropic Medicines Utilization Patterns in  the Treatment of Mental and Neurological Disorders in Public Hospitals of Hadiya Zone, Southern Ethiopia: A Record-based study

PMEN-D-26-00125R1

Dear Mr. Tirore,

We are pleased to inform you that your manuscript 'Assessment of Psychotropic Medicines Utilization Patterns in  the Treatment of Mental and Neurological Disorders in Public Hospitals of Hadiya Zone, Southern Ethiopia: A Record-based study' has been provisionally accepted for publication in PLOS Mental Health.

Before your manuscript can be formally accepted you will need to complete some formatting changes, which you will receive in a follow up email. A member of our team will be in touch with a set of requests.

Please note that your manuscript will not be scheduled for publication until you have made the required changes, so a swift response is appreciated.

IMPORTANT: The editorial review process is now complete. PLOS will only permit corrections to spelling, formatting or significant scientific errors from this point onwards. Requests for major changes, or any which affect the scientific understanding of your work, will cause delays to the publication date of your manuscript.

If your institution or institutions have a press office, please notify them about your upcoming paper to help maximize its impact. If they'll be preparing press materials, please inform our press team as soon as possible -- no later than 48 hours after receiving the formal acceptance. Your manuscript will remain under strict press embargo until 2 pm Eastern Time on the date of publication. For more information, please contact mentalhealth@plos.org.

Thank you again for supporting Open Access publishing; we are looking forward to publishing your work in PLOS Mental Health.

Best regards,

Karli Montague-Cardoso

Staff Editor

PLOS Mental Health

***********************************************************

Reviewer Comments (if any, and for reference):
